# Randomised phase-2 screening trial of intermittent energy restriction plus resistance exercise versus resistance exercise alone during chemotherapy for advanced breast cancer

**DOI:** 10.1038/s41416-025-03129-8

**Published:** 2025-07-31

**Authors:** Michelle Harvie, Mary Pegington, Anthony Howell, Yit Lim, Karen Livingstone, Danielle Rose, Debbie McMullan, Anthony Maxwell, Emma Barrett, Katharine Sellers, Suzanne Krizak, Sacha J. Howell

**Affiliations:** 1 Nightingale/Prevent Breast Cancer Centre, Wythenshawe Hospital, Manchester University NHS Foundation Trust, Manchester, M23 9LT UK; 2 Division of Cancer Sciences, The University of Manchester, Manchester, UK; 3 Manchester Breast Centre, Oglesby Cancer Research Centre, The Christie NHS Foundation Trust, University of Manchester, Manchester, UK; 4 Research and Innovation, Manchester University Hospital Foundation NHS Trust, Manchester, UK; 5 Centre for Biostatistics, The University of Manchester, Manchester, UK; 6 Department of Medical Oncology, The Christie NHS Foundation Trust, Manchester, UK

**Keywords:** Outcomes research, Breast cancer

## Abstract

**Background:**

Weight control and energy restriction could improve survival in patients with advanced breast cancer (ABC) but randomised data are lacking. A randomised screening trial was conducted to assess an intermittent energy restricted diet and resistance exercise intervention (IER + RE) vs RE alone (RE) on progression free survival (PFS), toxicity and Quality of Life (QoL) during chemotherapy for ABC.

**Methods:**

Sixty-eight women were randomised to IER + RE (*n* = 35) or RE (*n* = 33) with one-sided significance assessed at the 20% threshold. The primary end point was PFS secondary endpoints included chemotherapy toxicity, weight change and QoL.

**Results:**

The adjusted hazard rate for progression comparing IER + RE vs RE was 0.729 (0.391–1.361) and the median PFS 42.0 vs 26.1 weeks respectively (*p* = 0.160). Toxicity was low and comparable between groups. Comparing IER + RE vs RE alone at cycle 3 the median (interquartile range) changes were: weight –1.8 kg (–4.2 to –0.7) vs +0.2 kg (–0.74, 2.59) (*p* < 0.001), FACT-B + 4.0 (–0.8, 11) vs +1.0 (–4.0, 4.0) (*p* = 0.031) and Hospital Anxiety Depression Score –2.0 (–3.5, +0.5) vs +1.0 (–2, 3.5) (*p* = 0.022).

**Conclusion:**

IER + RE improved PFS and QoL without evidence of harms warranting a further larger randomised study in ABC.

**Trial registration:**

https://www.isrctn.com/ISRCTN12841416.

## Background

Excess adiposity and reduced lean body mass have been linked to reduced survival [1] and quality of life (QoL) [2] in women with advanced breast cancer (ABC). Preclinical studies in rodents have demonstrated reduced growth of advanced cancer with energy and carbohydrate restriction and weight reduction [3]. This is thought to be mediated by reduced insulin-related signalling and reduced Phosphoinositide 3-kinase (P13K), AKT, mammalian target of rapamycin complex 2 (mTOR2) signalling, reducing available nutrients to cancer cells, as well as reduction in other adiposity related factors such as adipokines and inflammation [3]. Energy and carbohydrate restriction and weight loss have the potential to improve cancer outcomes in patients with advanced disease. To date trials of energy restricted diets have been mainly small feasibility studies demonstrating short term adherence and potentially beneficial changes in biomarkers, with minimal outcome data. One potentially effective way to achieve energy and carbohydrate restriction and weight loss in patients with breast cancer is an intermittent energy restricted (IER) diet. We have previously shown, in the Breast-Activity and Healthy Eating after Diagnosis 2 (B-AHEAD 2) study that an IER diet (2 days of a low energy low carbohydrate intake/ week and 5 days of healthy Mediterranean diet) is associated with better reductions in weight and body fat than daily energy restriction, with a trend for reduced taxane chemotherapy toxicity in women receiving adjuvant treatment [4]. The B-AHEAD 3 study, reported here, aimed to test the effect of IER on progression free survival (PFS) and toxicity in women commencing a new line of chemotherapy for ABC. Participants in the test IER dietary group (IER + RE) and the control group (RE only) undertook the same resistance exercise programme to minimize reductions in lean body mass that has been associated with poorer outcomes [5].

## Methods

### Study design

We conducted a multi-centre two-arm randomised controlled phase-2 screening trial [6] of IER + RE vs. RE alone with standard written diet advice amongst women diagnosed with ABC. The study was designed to see whether IER could be beneficial, to provide an estimate of effect size and whether IER should be tested in a definitive phase 3 study. The study is written in accordance with CONSORT reporting guidelines (Supplementary Table 1).

### Participants

We included women with histologically confirmed locally advanced breast cancer that was not amenable to curative surgical resection or with metastatic breast cancer (MBC) measurable or non-measurable disease by RECIST v1.1, BMI ≥ 24 kg/m^2^ and performance status 0 or 1. Full eligibility is described in Supplementary Table 2.

### Recruitment

Women were invited to the study by their oncology team or research nurses before or shortly after commencing a new line of chemotherapy in 13 breast units in England (Supplementary Table 3). Posters and leaflets in patient areas were used to raise awareness of the study.

### Randomisation and stratification

Randomisation was 1:1 and was undertaken by an independent administrator using a computer minimisation programme held at the trial coordinating centre (The Nightingale Centre at Wythenshawe Hospital, Manchester University NHS Foundation Trust), stratifying for six factors:

1. First or second line chemotherapy vs. third or greater line chemotherapy
2. Paclitaxel/docetaxel containing vs. capecitabine containing vs. other
3. Above or below the projected median BMI of our cohort (BMI ≥ 30 kg/m^2^ vs. BMI < 30 kg/m^2^)
4. Positive or negative ER receptor status
5. Combination therapy vs. single agent chemotherapy
6. HER2 receptor positive or negative

### Study interventions

The IER + RE and RE only interventions are described according to the TIDieR checklist Supplementary Table 4. The diet intervention was delivered by specialist dietitians and the exercise intervention by physiotherapists and cancer exercise specialists with all staff having expertise and training in the management of metastatic breast cancer. The schedule of events for intervention delivery in the two study groups is reported in Supplementary Table 5.

### IER + resistance exercise

#### IER diet

 The diet was designed to provide an overall 25% energy restriction across the week to promote weight loss. Women were asked to follow a low calorie diet for two consecutive days/week throughout the trial. A typical low calorie day is shown in Supplementary Table 6. Participants receiving IV chemotherapy were asked to do the two low energy days immediately prior to the chemotherapy infusion, ideally starting at the first cycle. The IER diet has been described previously [4]. Energy restricted days limit to around 1000 kcal and ~50 g carbohydrate per day and includes lean meat, fish, eggs, vegetable protein sources i.e. tofu, textured vegetable protein, five portions of vegetables, one portion of fruit, 3 portions of low fat dairy foods or dairy alternatives, and some monounsaturated fat (MUFA). Women were asked to follow an energy restricted healthy Mediterranean type of diet for the remaining five days of the week. Energy intake on these days was tailored to their estimated energy requirements to provide an overall 25% energy restriction across the week as described previously [4]. Baseline energy requirements were predicted for each participant using Henry equations [7] multiplied by their reported activity level in metabolic equivalents [8]. The Mediterranean diet provides 30% energy from fat (15% monounsaturated fatty acids, 8% polyunsaturated fatty acids, 7% saturated fatty acids), 25% energy from protein and 45% from low glycaemic load carbohydrate, includes at least five portions of vegetables and two portions of fruit per day and is limited in alcohol (<10 units, 80 g per week).

Individualised diet advice was given as either a face-to-face or phone consultation with a trial dietitian. The consultations covered appropriate food choices, portion sizes, menu ideas, recipes, using appropriate behavioural techniques to enhance dietary adherence [9]. Participants also received a booklet which including guidance on dealing with chemotherapy related side-effects which can affect dietary intake such as nausea, dry and sore mouth, constipation or diarrhoea, food hygiene and which discouraged the use of self-prescribed nutritional supplements. Three-weekly dietitian phone reviews checked adherence, reinforced the dietary goals and provided individualised trouble shooting, followed by an email summary of the key issues. Participants received healthy tips sheets every 3 weeks which covered topics to increase adherence to the energy restricted diet (see Supplementary Table 6: Schedule of events for intervention delivery in the two study groups, and the TIDieR checklist are reported in supplementary Tables 4, 6).

##### Resistance exercise

Participants received face to face advice to follow a personalised home-based RE programme advice from one of the trial physiotherapists at Manchester University NHS Foundation Trust or within the local unit for patients recruited in Plymouth. The programme had been adapted from Cormie et al. [10] and included three self-supervised sessions per week. Each session started with a brief warm up, then 5–10 standard exercises for muscle groups in the lower limbs, upper limbs, trunk then a cool down (Supplementary Table 7). The programme was individualised for patients according to the site of any metastases, co-morbidities and current activity and energy levels. All participants had an initial one to one session with a trial physiotherapist to practice the correct technique and allow the physio to adapt the programme, e.g. performing the exercises seated or lying instead of standing. Participants with lymphoedema were advised to wear their sleeve when undertaking upper body exercise. Participants were provided with online demonstration videos (Physiotec, Canada www.physiotec.ca) and paper instructions for their recommended exercises. Patients were also given general guidance to include 150 min of moderate (not vigorous) activity per week as per standard advice for cancer patients receiving chemotherapy and for general health. Participants received a review phone call from the trial physiotherapist or cancer exercise specialist every three weeks to assess their progress with resistance exercise and check that they are using the correct technique. Exercise progression (increasing the number of repetitions or increasing the weight used) took place at the discretion of the trial physiotherapists and exercise specialist (Supplementary Tables 4,6).

#### Resistance exercise group (standard written diet advice + resistance exercise)

Women received the identical resistance exercise programme to the IER + RE group including 3 weekly physiotherapy/ exercise specialist review calls. Women received standard written leaflets advising them of the potential importance of weight loss, how to follow a healthy Mediterranean diet and information about nutritional supplements, food hygiene and chemotherapy-related side effects. They did not receive dietetic advice or support (Supplementary Table 6).

#### End of study

Women continued with their allocated IER + RE or RE programmes until disease progression. If chemotherapy was discontinued due to toxicity or at the planned end of the treatment course, participants continued with the allocated programme. Women who wanted to discontinue their allocated programme were asked for permission for the research team to monitor their CT scans until disease progression, at which time participants left the study.

##### Primary outcome measure

The primary outcome was PFS, with progression defined according to RECIST v1.1 (Response Evaluation Criteria in Solid Tumours) guidelines [11] assessed by radiologists blinded to group allocation. CT scans were performed as per local protocols in the chemotherapy centres, usually every 3–4 cycles, i.e. around every 9-12 weeks and RECIST v1.1 was assessed by radiologists in the recruiting centres. Scans were also assessed centrally using RECIST v1.1 by study radiologists at the trial coordinating centre. PFS was calculated for both local and central assessments. Central assessment was initially planned as the primary endpoint; however, we were unable to access final scans for 12 patients so local assessments are presented as the primary outcome.

##### Secondary outcome measures

Change in the following end points were assessed using standardised methods in the recruiting centres as per the schedule in Supplementary Table 8: Chemotherapy toxicity using CTCAE v4 [12], and taxane neuropathy using an automatic vibration assessment tool by identification of vibration/ no vibration on the index finger and big toe (VibraTip,McCallan Medical Ltd, Northamptonshire). Chemotherapy dose reductions and delays were recorded. Dose reductions were recorded from the starting dose where the starting dose had been chosen by the treating oncologist as standard of care.

Visceral and subcutaneous fat and lean body mass at the transverse processes of the third lumbar vertebrae(L3) were assessed using commercial image analysis software (Analyze 12.0 - AnalyzeDirect Inc., Kansas, USA) from the routine monitoring CT scans. Software segmentation was performed and the cross-sectional area of skeletal muscle (including psoas, paraspinal muscles and the abdominal wall muscles) was measured together with the area of visceral abdominal fat (the fat lying within the abdominal cavity) and the deep and superficial subcutaneneous fat (the fat superficial to the abdominal wall lying deep to and superficial to the deep fascia respectively). Abdominal muscle mass was used to estimate total lean body mass in advanced cancer patients using the equation described by Mourtzakis et al. [13] (LBM (kg) = 0.30 x [skeletal muscle at L3 using CT (cm2)] + 6.06).

The CT assessment of muscle at L3 will not detect any potential changes in lean body mass in the upper and lower limbs. We therefore included functional assessments of lower limb strength (five repetition sit to stand normalised by weight sit to stand test) [14] and upper limb strength using hand grip dynamometer tests [15]. We assessed quality of life FACT-B, FACT-ES [16] FACT-F [17], FACT-BP [18], hospital anxiety and depression score (HADS) [19] in all participants and FACT-Taxane for participants on taxane chemotherapy [20]. We assessed adherence to the two low energy days in the IER + RE group and adherence to the resistance exercise in both group using trial specific forms. In both groups we also assessed adherence to the Mediterranean diet using a validated 12-point Mediterranean diet score [21] and amount of self–reported cardiovascular exercise using the Scottish Physical Activity score [22]. Fidelity of delivery of the diet and exercise interventions was assessed in both groups through receipt of dietitian and physiotherapy calls. Time-to-treatment-failure (TTF) was also measured, with treatment failure defined as either progression or discontinuation of chemotherapy due to toxicity.

### Statistics

The sample size was calculated for a phase II screening design [6], based on a primary outcome of PFS, with a time-to-event hazard ratio (HR) of 0.65, and with 90% power and a one-sided significance of 20%. With an estimated a PFS of 5.4 months in the RE arm and a 15 month accrual period and 15 month follow-up, a total of 98 events were required overall. This corresponded to 114 patients required and allowing for a 15% drop out our recruitment target was 134 patients.

Continuous outcomes are reported as median and interquartile range (IQR), and categorical outcomes as counts and percentages (%). Comparisons between groups are made by Wilcoxon rank-sum test, and chi-square test, or Fishers Exact test, where appropriate. Time-to-event outcomes, PFS and TTF, were compared between groups, using Cox-proportional hazards regression, with and without adjustment for presence of visceral metastasis, in all subjects recruited (intention to treat analysis). For the primary outcome PFS, a per protocol sensitivity analysis was performed, excluding those who had not received their allocated diet or exercise intervention. We report the number of time points with CTCAE toxicity > or = grade 1, or with severe toxicity (> or = grade 3), and frequency of each grade of toxicity for the key toxicities including neuropathy (assessed with VibraTip), across all cycles of chemotherapy in each group. The secondary outcomes (toxicity, neuropathy, changes in fat and lean body mass, quality of life [QOL] and muscle strength) were assessed at each cycle, with formal comparison between groups of changes from baseline to the start of cycle 3 only. The primary analysis is one-sided with 20% significance level, and all secondary analyses two-sided with 5% significance level. All analyses were performed using R statistical software v4.3.0 or later.

### Blinding

Staff assessing key trial endpoints were blinded to study arm, i.e., radiology staff assessing RECIST and change in fat and muscle area in CT scans, and researchers analysing the QOL questionnaires.

### Safety considerations

If BMI decreased to <19 kg/m^2^, participants in the IER + RE group were advised to increase calories on the non-restricted days, whilst maintaining the IER diet. If lean body mass was reducing and sarcopenia was developing (defined as lumbar skeletal muscle index <38.5 cm^2^/m^2^ from CT scans [5]) diet and exercise advice were adjusted to try to limit further reductions. Since weight loss could have resulted in increased toxicity, participants who lost >10% of their body weight would have their chemotherapy dose reduced if they had experienced grade ≥2 toxicity. If progression of bone metastases prevented safe resistance exercises, participants were advised to discontinue the study exercises but continue with the other aspects of the study.

We recorded any serious adverse events [12] during the study, but excluded a predefined list of events which were associated with delivering chemotherapy to women with advanced breast cancer and unrelated to the diet and exercise programme.

## Results

**Fig. 1:**
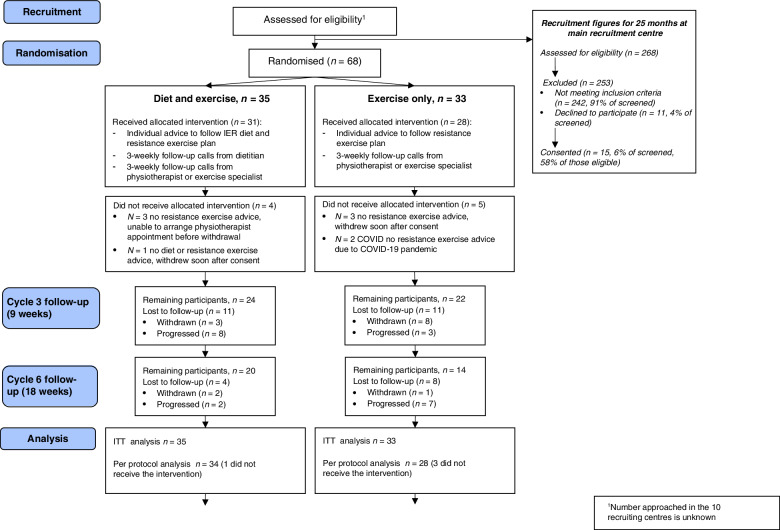
Consolidated standards of reporting trials (CONSORT) for the B-AHEAD-3 trial.

Sixty-eight women were recruited from May 2015 until the study closed during the COVID pandemic in March 2020. One of the IER + RE group did not receive diet or exercise advice (3%) and four had received their dietary intervention but did not receive exercise advice (11%), while five of the RE group (15%) did not receive their allocated exercise advice, two of these due to COVID. Five of the IER + RE (14%) and 9 of the RE group (27%) withdrew (Fig. 1 Consort diagram). Progression free survival was censored on 30^th^ March 2020 at which time 3 patients in the IER + RE group were still active in the trial.

Groups were well matched for demographics, type of breast cancer and treatments. Mean (SD) age was 58.0 (9.9) years and median (95% CI) BMI was 29.8 kg/m^2^ (27.0–31.6). Fifty-eight (85%) had ER positive and 19 had (13%) HER2 positive disease. Forty-four (65%) were receiving first line chemotherapy treatment, 37 (54%) were receiving capecitabine and 22 (32%) taxane based chemotherapy (Table 1).

**Table 1:** Characteristics of participants randomised to the IER + RE and RE groups.

	IER + RE (*n* = 35)	RE (*n* = 33)	All *n* = 68
**Age**	57.5 (10.6)^a^	58.5 (9.2)	58 (9.9)
**Weight (Kg)**	78.0 (73.0–84.8)^b^	75.0 (70–88.8)	77.0 (71.0–85.8)
**BMI- kg/m2**	29.2 (26.4–31.3)^b^	30.0 (27.0–32.1)	29.8 (27.0–31.6)
**Current Chemotherapy type**^c^			
Taxane	11 (31)	11 (33)	22 (32)
Capecitabine	18 (51)	19 (58)	37 (54)
Other	6 (17)	3 (9)	9 (13)
**Previous breast cancer treatment n(%)**			
Adjuvant or neoadjuvant chemotherapy	20 (57)	18 (55)	38 (56)
Metastatic endocrine	21 (60)	25 (76)	46 (68)
**Family history BC**^c^	14 (45)	13 (50)	27 (47)
Unknown	4	7	11
**ER Positive**^c^	29 (83)	29 (88)	58 (85)
**HER Positive**^c^	5 (14)	4 (12)	9 (13)
**De novo metastatic**	8 (23)	13 (39)	21 (31)
**Site of metastases**^c^			
Bone	15 (43)	19 (58)	34 (50)
Liver	21 (60)	21 (64)	42 (62)
Lung*	10 (29)	11 (33)	21 (31)
Brain or leptomeningeal	2 (6)	2 (6)	4 (6)
Lymph node	16 (46)	12 (36)	28 (41)
Colon only	1 (3)	–	1 (1)
Locally advanced/ distal disease	4/31	2/33	6/68
**Line of metastatic chemo**^c^			
1	24 (69)	20 (61)	44 (65)
2+	11 (31)	13 (39)	24 (35)
Menopause**^c^			
Pre (“still having regular periods”)	5 (14)	3 (9)	8 (12)
Post (“not having regular periods”)	30 (86)	30 (91)	60 (88)
**Smoking status**^c^			
1 Current	1 (3)	2 (8)	3 (5)
2 Never	16 (52)	17 (65)	33 (58)
3 Ex	14 (45)	7 (27)	21 (37)
Unknown	4	7	11
**Ethnicity**^c^			
White	34 (97)	32 (97)	66 (97)
Black/Mixed	1 (3)	1 (3)	2 (3)
**Deprivation quintile**^c^			
1 (Most deprived)	3 (9)	5 (15)	8 (12)
2	5 (14)	10 (30)	15 (22)
3	4 (11)	2 (6)	6 (9)
4	10 (29)	4 (12)	14 (21)
5 (Least deprived)	12 (34)	12 (36)	24 (35)
**Comorbidities**^c^			
Cardiac	3 (9)	2 (6)	5 (7)
Type 2 Diabetes	1 (3)	1 (3)	2 (3)
Gastric	2 (6)	2 (6)	4 (6)
Musculoskeletal	2 (6)	2 (6)	4 (6)
Neurological	3 (9)	1 (3)	4 (6)
Respiratory	2 (6)	-	2 (3)
Thyroid	2 (6)	-	2 (3)
None	21 (60)	26 (79)	47 (69)

*One participant had ovarian metastases in addition to lung a mean(sd) b median(IQ range) c n(%).

**Participants were considered post-menopausal if they were ≥55 years at entry, otherwise they were considered pre/perimenopausal based on criteria by Phipps et al. [52]

### Primary outcome

The intention to treat analysis showed a significantly lower hazard rate for progression in the IER + RE group compared to REs after adjusting for confounders, with one-sided Wald test at the 20% threshold. Unadjusted analyses showed a 36% decrease in progression in the IER + RE group (HR [95% CI] 0.635 [0.356–1.135], *p* = 0.0625). After adjusting for presence of visceral metastases, the IER + RE group had a 27% lower chance of progression compared with the RE group (HR [95% CI] 0.729 [0.391–1.361], *p* = 0.160).

**Fig. 2:**
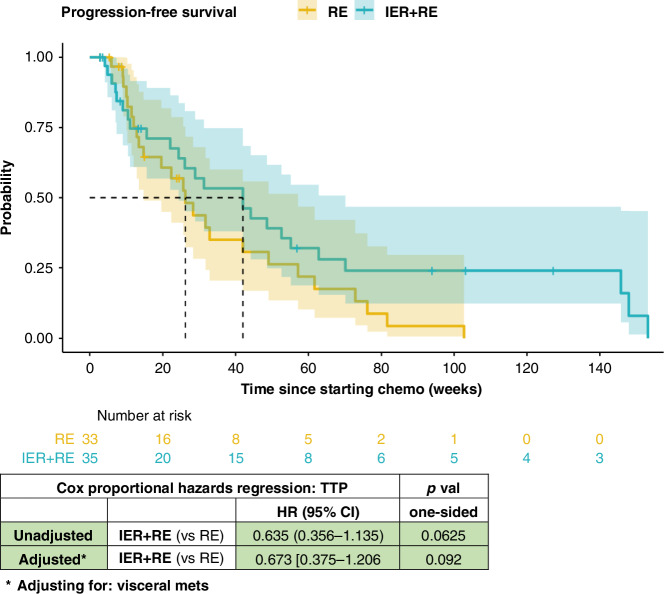
Kaplan-Meier estimates of progression-free survival in the intention-to-treat population for IER + RE vs RE. Progression free survival of IER + RE vs RE adjusting for: visceral metastasis.

Figure 2 shows the separation of the two groups with the IER + RE group reaching a 50% chance of progression at 42.0 weeks compared with 28.3 weeks for the RE group (*p* = 0.160).

### Secondary outcomes

#### Progression free survival determined by centrally read CT scans

Fifty-six patients were included in the analysis, 29 in the IER + RE group and 27 in RE. The remaining patients (6 IER and 6 RE) were not included as the local centre had determined clinical progression and withdrew patients from the study despite imaging suggesting stable disease. The hazard ratios for the effects of IER + RE on progression free survival in this analysis were; unadjusted IER + RE vs RE (HR 0.568, 95% CI 0.295–1.094) (*p* = 0.045) and in the analyses adjusted as above (HR 0.601, 95% CI 0.294–1.229) (*p* = 0.081), comparable to the analyses using local assessment of progression.

#### Progression free survival per protocol analysis

Per protocol analysis of IER + RE vs RE which only included subjects who had received their initial allocated diet or exercise advice intervention (IER + RE *n* = 35, RE *n* = 31) showed similar results to the main intention to treat analysis with an unadjusted HR 0.661 (0.366–1.193), *p* = 0.083 and adjusted HR 0.752 (0.399–1.417), *p* = 0.186.

#### Chemotherapy toxicity

Presence of any key CTCAE assessed toxicities (peripheral neuropathy, palmar plantar erythrodysesthesia, diarrhoea, nausea, vomiting, neutropenia, fatigue) were similar in both groups. During the first 16 cycles the median (IQR) time points with toxicity measurements recorded were: IER + RE 4 (2–6) and RE 2 (1–8). The median (IQR) percentage of time points when participants reported experiencing one or more key toxicities of any grade was 100 (IQR 77–100) % for IER + RE and 100 (73–100) % for RE (*p* = 0.923). Very few participants experienced severe (≥ grade 3) toxicities in the two groups. The median (range) percentage of time points with severe toxicity were IER + RE 0 (0–1) % and RE 0 (0–1) % in the first 16 cycles (*p* = 0.447). The numbers with grade 1, 2 and 3 toxicity for key chemotherapy toxicities is reported in Table 2. Presence of neuropathy measured by VibraTip in participants receiving taxane-based chemotherapy (*n* = 22) was also similar in the two groups. The percentage of time points where neuropathy was observed were median (IQR) 0 (0–46) % for IER + RE and 0 (0–31) % for RE (p = 0.566).

**Table 2:** Numbers with chemotherapy toxicity for the IER + RE and RE groups across all chemotherapy cycles.

	IER + RE (*n* = 35)				RE (*n* = 33)			
Toxicity	None	Grade 1	Grade 2	Grade 3	None	Grade 1	Grade 2	Grade 3
Diarrhoea	19 (59.4)	8 (25)	2 (6.2)	3 (9.4)	16 (55.2)	13 (44.8)	*–*	*–*
Fatigue	6 (19.4)	17 (54.8)	8 (25.8)	*–*	3 (10.3)	18 (62.1)	8 (27.6)	*–*
Febrile neutropenia	30 (100)	*–*	*–*	*–*	26 (96.3)	*–*	*–*	1 (3.7)
Nausea	17 (53.1)	10 (31.2)	5 (15.6)	*–*	17 (58.6)	12 (41.4)	*–*	*–*
Palmar-plantar erythrodysesthesia	17 (54.8)	11 (35.5)	2 (6.5)	1 (3.2)	13 (44.8)	13 (44.8)	3 (10.3)	*–*
Peripheral sensory neuropathy	16 (50)	14 (43.8)	2 (6.2)	*–*	15 (51.7)	14 (48.3)	*–*	*–*
Vomiting	26 (81.2)	5 (15.6)	1 (3.1)	*–*	27 (93.1)	2 (6.9)	*–*	*–*

Chemotherapy dose reductions and delays were reported in 2/35 (6%) and 3/35 (3%), respectively for IER + RE and 1/33 (3%) and 4/33 (12%), respectively for RE.

#### Weight and body composition

Changes in weight and body composition are reported in Table 3. At cycle 3, weight change in the IER + RE group was median –1.8% (–4.2 to –0.7) compared with +0.2% (–0.74 to 2.59) in RE. More participants achieved a clinically significant weight loss of 5% in the IER + RE group. At cycle 3, 4 participants (12.5%) had achieved 5% weight loss in the IER + RE group compared with 0 participants in the RE group.

**Table 3:** Change in body weight, abdominal fat and muscle stores between baseline and cycle 3 (week 9 of the trial) in the IER + RE and RE groups.

Change between baseline and cycle 3	IER + RE (*n* = 35)	RE (*n* = 33)	All *n* = 68	*P* value IER + RE vs RE
***N***	*n* = 30	*n* = 30	*N* = 60	
Body weight (kg)	–1.50 (–2.78, -0.60)	0.10 (–0.60, 1.90)	–0.70 (–1.94, 0.13)	<0.001
% Change	–1.78 (–4.19, -0.72)	0.15 (–0.74, 2.59)	–0.87 (–2.33, 0.18)	
VAT mm^2^	–1002 (–2041, 330)	–378 (–1905, 1180)	–582 (–1991, 984)	0.143
% Change	–7.9 (–17.5, 7.9)	–3.2 (–12.7, 10)	–4.7 (–15.4, 9.2)	
DSAT mm^2^	–751 (–1826, 111)	–14 (–1336, 1497)	–446 (–1530, 635)	0.232
% Change	–6.2 (–13.3, 0.9)	–0.2 (–10, 9.8)	–3.9 (–12.5, 6.0)	
SSAT mm^2^	–870 (–2279, 495)	285 (–1481, 1446.)	–332 (–1852, 860)	0.115
% Change	–3.8 (–19.7, 7.7)	2 (–6.2, 7.4)	–3 (–16.4, 7.6)	
Total SAT mm^2^	–1568 (–3452, 658)	–220 (–2256, 2356)	–994 (–2921, 1680)	0.281
% Change	–5.5 (–13.5, 2.1)	–0.9 (–5.4, 9.1)	–3.4 (–8.4, 5.7)	
Total AT mm^2^	–2454.5 (–4233, 987)	276 (–3475, 3502)	–1454 (–4091, 2066)	0.176
% Change	–6.6 (–11.8, 2.3)	0.8 (–8.2, 8.8)	–3.7 (–9.4, 5.2)	
Muscle mm^2^	–59 (–560, 217)	–153.5 (–587, 502)	–72 (–574, 285)	0.694
% Change	–0.6 (–5.4, 1.9)	–1.4 (–5.6, 4.4)	–0.8 (–5.6, 2.3)	
Skeletal muscle index	–0.22 (–2.13, 0.75)	–0.57 (–2.33, 1.80)	–0.26 (–2.21, 1.06)	0.658
% Change	–0.6 (–5.4, 1.9)	–1.4 (–5.6, 4.4)	–0.8 (–5.6, 2.3)	

Median (interquartile range).

*VAT* visceral adipose tissue, *DSAT* deep subcutaneous fat, *SSAT* superficial subcutaneous fat, *SAT* subcutaneous tissue, *AT* abdominal tissue.

There was wide variation in change in body composition during the study across both groups. At cycle 3 fat stores appeared to decrease more in the IER + RE than the RE group. For example, median (IQR) change in visceral adipose tissue was –1002 (–2041 to +330) mm^2^ in the IER + RE group and –378 (–1905 to + 1180) mm^2^ in the RE group (*p* = 0.143). Change in total subcutaneous fat was –1568 (–3452 to +658) mm^2^ in the IER group and –220 (–2256 to +23456) mm^2^ in the RE group (*p* = 0.281). Changes in muscle area were comparable between the two groups; median (IQR) change in the IER + RE group was –59 (–560 to + 217) mm^2^ compared with –153 (–587 to +502) mm^2^ in the RE group (*p* = 0.694).

#### Muscle strength

Change in arm and leg strength as measured by hand grip dynamometer and five repetition sit-to-stand tests at cycle 3 were similar in both groups. Performance time for the five repetition sit-to-stand test increased by a median of 0.4 s (IQR 0.9–1.5) for IER + RE compared with –0.3 s (–1.3 to 0.5) for RE (*p* = 0.433). Arm strength showed a very small median increase in both groups; median (IQR) 0.3 (IQR –1.3 to 2.6) kg IER + RE group and 0.3 (–2.2 to 1.7) kg RE, *p* = 0.598 (Supplementary Table 9).

#### Quality of life

Changes in quality of life at cycle 3 are reported in Table 4. Median (IQR) FACT–B, increased more in the IER + RE than the RE group indicating an improved quality of life; +4.0 (–0.8, 11.0) IER + RE, +1.0 (–4.0, 4.0) RE (*p* = 0.031). Similar trends were also seen for FACT-ES and FACT-G, and FACT-taxane, while HADS decreased more in the IER + RE than the RE group –2.0 (–3.5 to +0.5) IER + RE and +1.0 (–2.0 3.5) RE (*p* = 0.022). In the 11 participants that completed the FACT-Taxane QoL questionnaires the IER + RE group there was a trend towards less reporting of subjective taxane toxicity (*p* = 0.066).

**Table 4:** Change in quality of life in the IER + RE and RE groups between baseline and cycle 3 (Week 9 of the trial).

Quality of life measure	IER + RE (*n* = 35)	RE (*n* = 33)	All *n* = 68	*p* value IER + RE vs RE
FACT	*n* = 27	*n* = 21	*n* = 48	
FACT-B	4.0 (–0.8, 11.0)	1.0 (–4.0, 4.0)	1.9 (–3.0, 9.2)	0.031
FACT-ES	6.0 (–1.9, 19.5)	1.0 (–3.0, 8.7)	3.5 (–3.0, 14.0)	0.157
FACT-G	4 (–4.7, 13.0)	–0.2 (–4.4, 4.0)	1.5 (–4.6, 11.5)	0.174
FACIT-F	2 (–2.5, 7.5)	2.0 (–2.0, 5.0)	2 (–2.3, 5.1)	0.744
FACT-Taxane *	*n* = 6	*n* = 5	*n* = 11	
	3 (0.62, 13.5)	–5.0 (–9.0, –2.0)	0.0 (–5.0, 2.8)	0.066
FACT-BP (Bone Pain)**	*n* = 10	n = 14	*n* = 24	
	2 (0, 3.8)	–3.0 (–5.8, 0.8)	0 (–4.3, 3.3)	0.205
HADS	*n* = 27	*n* = 23	*n* = 50	
	-2 (-3.5, 0.5)	1.0 (–2.0, 3.5)	–1.0 (–3.0, 2.0)	0.022

*FACT-B* Functional Assessment of Cancer Therapy—Breast, *FACT-ES* Functional Assessment of Cancer Therapy—Endocrine scale, *FACIT* Functional Assessment of Chronic Illness Therapy—Fatigue Scale, *FACT-G* Functional Assessment of Cancer Therapy—General, *FACT-BP* Functional Assessment of Cancer Therapy—Bone pain, *FACT-T* Functional Assessment of Cancer Therapy—Breast Taxane, *HADS* Hospital Anxiety and Depression Scale.

*Only patients receiving taxanes.

**Only patients with bone metastases.

#### Dietary adherence

Data from all participants in the IER + RE group showed they completed median (IQR) 75 (38–92) % of their expected low calorie days during their chemotherapy treatments which lasted median (IQR) 10.0 (5.8–19.2) weeks. This decreased to 37.5 (2.5–73.1) % of their expected low calorie days in the period on the study after chemotherapy had finished, which lasted median (IQR) 23.5 (7–57) weeks. Mediterranean diet scores were available for a sub-set of participants in both groups at both baseline and chemotherapy cycle 3 *n* = 16 IER + RE and *n* = 12 RE. Median (IQR) baseline scores out of a possible 12 point score were IER + RE 4.5 (3–6.0) RE3.5 (2.3–6.8). At cycle 3 there was a modest increase in score in the IER + RE group median (IQR) change was 2.5 (1.3–3.8). Scores were unchanged in the RE group –0.5 (–1 to +1.5).

#### Resistance exercise adherence and physical activity

The resistance exercise adherence questionnaire was completed by *n* = 24 IER + RE and n = 20 RE participants at cycle 3. At cycle 3 both groups reported median 45 min resistance exercise per week (IQR for IER + RE 0–90, RE 23–95) minutes. Both groups reported a decrease in cardiovascular PA duration from baseline to cycle 3 (Supplementary Table 10).

### Delivery of the interventions

Most of the IER + RE group received their scheduled three weekly diet calls during chemotherapy with median (IQR) calls received 95% (58–100), and 71% (50–88) after chemotherapy. Three weekly physiotherapist/exercise specialist calls during chemotherapy were received by median (IQR) 67% (50–86) in the IER + RE group and 76% (61–100) in the RE group. After chemotherapy, the calls were received by 54% (43–100) of the IER + E group and 0% (0–80) of the RE group. Over the whole study the IER + RE group received 84% (57–100) of their diet calls and 63% (50–81) of their physiotherapist/exercise specialist calls compare to 72% (52–88) in the RE group.

### Time to treatment failure

Time to treatment failure (either progression or discontinuation of chemotherapy due to toxicity) was comparable between the two groups (Supplementary Table 11 and Supplementary Fig. 1). Time to treatment failure in the IER + RE group compared with the RE group: HR (95% CI) 0.695(0.389–1.241), *p* = 0.219.

### Serious adverse events

There were no serious adverse events reported in either group which were considered to be related to the diet or resistance exercise interventions.

## Discussion

The study shows potential benefits of the studied IER regimen for progression free survival in patients with advanced breast cancer receiving chemotherapy. The IER diet did not impact on chemotherapy toxicity which was relatively low in both groups. Self-reported data showed good adherence to the IER diet which was associated with modest reductions in weight and abdominal fat stores, alongside improvements in quality of life scores.

This is one of the few trials to report the effect of diet and exercise on cancer outcomes amongst patients with breast cancer. The studied IER intervention includes a number of components which may impact on outcome i.e. weight loss, intermittent fasting and a Mediterranean diet. The intentional weight loss alongside an IER diet and resistance exercise programme in the B-AHEAD 3 study was not associated with harm. This contrasts to the worse outcomes reported in observational studies, with what is presumed to be unintentional weight loss in sicker cancer patients, resulting in reverse causality [23]. A recent randomised trial reported greater tumour response in women receiving a low carbohydrate / low energy IER + RE alongside an 8% weight loss in women with locally advanced breast cancer but not in those with distant metastases [24]. The DIRECT study in the neoadjuvant setting, in women with early breast cancer, did not shown a benefit of a fasting mimicking diet (FMD) (3 days of low calorie, low carbohydrate and protein before each chemotherapy cycle) for achieving pathological complete response in women with stage II–III HER2-negative breast cancer [25]. Potential diet effects in this study may have been limited by low diet adherence (only 34% of patients completed at least 4 FMD cycles) and minimal weight reduction (~1%) [26]. In contrast the Leaner Study in the neoadjuvant setting reported increased rates of pathological complete response with a combined cardiovascular resistance exercise and healthy diet programme compared to a control group (53% v 28%; *P* = 037) [27]. However, the beneficial effects may have been linked to the exercise intervention as there were minimal dietary changes and no difference in weight loss between the groups.

The weight independent effects of the Mediterranean diet on cancer outcome in this study are not known but are likely to be minimal. Current evidence suggests adherence to a Mediterranean diet has beneficial effects for overall mortality and non-cancer outcomes amongst patients with breast cancer. However, there is limited evidence for direct effects on cancer outcomes i.e. recurrence, cancer specific mortality [28]. Similarly, there is a low certainty of evidence supporting Mediterranean diet adherence and risk of breast cancer from observational studies [29]. The highly cited PREDIMED cardiovascular prevention randomised trial reported reductions 68% reductions in risk of breast cancer in women as a secondary endpoint amongst the group including high intakes of a polyphenol enriched olive oil but no other changes to weight or dietary composition [30]. Breast Cancer was a secondary endpoint of the PREDIMED study, and the study only had a 15% power for this endpoint, hence the point estimate has low credibility [31]. This notwithstanding the IER group in the current study only had modest improvements in their Mediterranean diet score which do not align with the high polyphenol olive oil intervention in the PREDIMED study.

In B-AHEAD 3 there was no reduction in chemotherapy toxicity with IER which contrasts with a trend for lower docetaxel toxicity in our previous B-AHEAD 2 study [4]. However, there were few reports of taxane neuropathy, perhaps reflecting less desire to maintain dose intensity in the ABC setting compared with the curative adjuvant setting. The majority of participants received well-tolerated weekly paclitaxel [32] or oral capecitabine regimens [33], although in the 11 participants that completed the FACT-Taxane QoL questionnaires there was a trend towards less reporting of subjective taxane toxicity in the IER + RE group. A recent systematic review concluded that there is no evidence that fasting regimens reduce chemotherapy associated toxicity [34].

There was a good self-reported adherence to the IER diet alongside chemotherapy treatment (median 75% of prescribed days) in patients with ABC, which is comparable to our previous report of 67% adherence amongst women with early breast cancer receiving adjuvant / neoadjuvant chemotherapy [4]. Khodabakhshi et al. reported 67% adherence to a low carbohydrate diet alongside chemotherapy amongst patients with advanced breast cancer [24].

There was a trend for reduced abdominal fat stores in the IER group. There was no overall change in abdominal fat in the RE group. Some subjects experienced increased, whilst other experienced decreased fat stores as previously described in a cohort of patients receiving paclitaxel for advanced breast cancer [35]. Reductions in adiposity may be important for improving cancer outcomes with IER. Lower composite fat scores (mean of visceral, subcutaneous, and intramuscular fat) have been associated with a better overall and disease-free survival in patients with locally advanced breast cancer [1]. Excess adiposity is thought to impact on the cancer microenvironment and increase production of leptin, plasminogen activator inhibitor and endothelial growth factor, reduced adiponectin, insulin resistance and inflammation [36]. There was no significant decrease in muscle area overall in either group, but there were increases and decreases amongst individuals in both groups, which has been previously reported [35]. We did not measure muscle attenuation (density). Low muscle attenuation is associated with accumulation of intramuscular fat accumulation and considered a better marker of cancer outcome than muscle area [37].

The greater improvements in FACT-B and HADS quality of life scores in the IER + RE group exceed minimum clinically significant differences of 2–3 points for FACT-B [38] and 1–2 for HADS [39] with similar sized trends in fatigue and endocrine symptoms. Trials of paclitaxel and capecitabine in patients with advanced breast cancer have showed stable quality of life during therapies [40, 41]. The quality of life improvements with the IER + RE group may in part be linked to greater health care attention they received than the RE groups as they received both dietitian and physiotherapist/exercise specialist calls every 3 weeks compared to the RE group who received one physiotherapist/exercise specialist call only every 3 weeks [42].

The strengths of this study include the comprehensive assessments of PFS, toxicity, body composition, adherence and quality of life. The collection of outcome data within recruiting centres at routine clinical appointments and CT scans allowed data capture without increasing trial burden on participants, and maximised outcome data collection. The diet and exercise IER+REs were delivered by specialist dietitians and physiotherapist/ cancer exercise specialists to maximise safe effective delivery to our target population, which likely maximised adherence to the IER + RE interventions.

There are several limitations to the study. We did not have screening figures for many of the recruiting sites. Recruitment to the study was slow and did not occur at all in some of the recruiting sites. Figures for the main recruiting site suggest a relatively small pool of patients had been informed about the study, most likely as patients were preferentially invited to trials of investigational medicinal products or gatekeeping by health care professionals reluctant to invite patients with advanced cancer to a diet and exercise study [43]. Once patients were invited to the study there appeared to be a good uptake (58% amongst those eligible) which is comparable to other diet studies amongst women with advanced breast cancer (53% amongst those eligible) [44]. There was good study retention (86% in the IER + RE group and 76% in the RE group). A recent overview of diet studies in cancer patients reported ~70% retention [45]. However, the study was closed before meeting the recruitment target meaning it was underpowered.

Secondary end points, i.e. body composition, chemotherapy toxicity, muscle strength, quality of life were assessed at each cycle while active in the trial. We report these secondary end points at cycle 3 to standardise these measures after different durations of chemotherapy but acknowledge these data do not inform long term effects of the diet when adherence is likely to decline. We had planned to report centrally scored RECIST but have used readings from the recruiting centres which will include variation that occurs with clinical measurements. The cohort recruited was mainly white (97%) but from a range of socioeconomic backgrounds. The lack of ethnic diversity in breast cancer research is a well-known issue. Further work is required to promote equity of inclusion in breast cancer research [46].

The potential role of nutrition and exercise on survival and quality of life have been identified as key unmet research priorities in a recent patient, caregiver and clinician priority setting partnership [47]. Ongoing diet studies are testing response to chemotherapy amongst women with advanced breast cancer including the DREAM study with 50 women randomised to an energy and carbohydrate restricted diet 48–72 h prior to each chemotherapy infusion and 30–60 min of moderate-intensity cycle ergometer exercise during each chemotherapy infusion, for up to six treatment cycles [48]. A low carbohydrate, low energy (ketogenic) diet is currently under study in China (*n* = 518) to determine its effects on chemotherapy sensitivity [49].

The results of the B-AHEAD 3 trial reported here suggest further trials of IER + RE in ABC are warranted. A future study would require ~350–375 patients to demonstrate a time to progression hazard ratio of 0.66, 90% power and two-sided significance of 5%. Such a study will require significant investment in time and energy to educate research centres to facilitate recruitment. Successful strategies for recruitment to exercise studies are likely to be effective for a planned diet and exercise study. These include training motivated designated health care professionals within the care pathway to undertake direct recruitment [50]. Successful recruitment is also likely to also require a designated funded recruiter [51].

## Supplementary information

supplemantary file

supplemantary file

## Data Availability

The datasets generated in the current study are available from the author on reasonable request.
