# Supplementary material for: Randomised phase-2 screening trial of intermittent energy restriction plus resistance exercise versus resistance exercise alone during chemotherapy for advanced breast cancer
Source: Br J Cancer. 2025 Jul 31;133(7):1010–9. doi: 10.1038/s41416-025-03129-8 (PMC12479916; doi:10.1038/s41416-025-03129-8)
Supplement: Supplementary file 1 — supplemantary file [file 41416_2025_3129_MOESM1_ESM.doc]

**Supplementary Information**

**Contents**

**Supplementary tables**

1. Consort Statement
2. Eligibility criteria
3. Recruiting hospital sites and numbers recruited from each site
4. Template for Intervention Description and Replication (TIDieR) checklist
5. Schedule of events for intervention delivery in the two study groups
6. Typical meal plan on the intermittent energy restriction (IER) diet
7. List of resistance exercises in the resistance exercise programme
8. Schedule of assessments for the two study groups
9. Change in leg & arm muscle strength from baseline (start of cycle 1) to cycle 3 (week 9) in the IER+RE and RE groups
10. Self-reported resistance and cardiovascular and resistance exercise in the two groups
11. Time to treatment failure in the IER+RE and RE groups

**Supplementary Figure**

1. Time to treatment failure in the IER+RE and RE groups


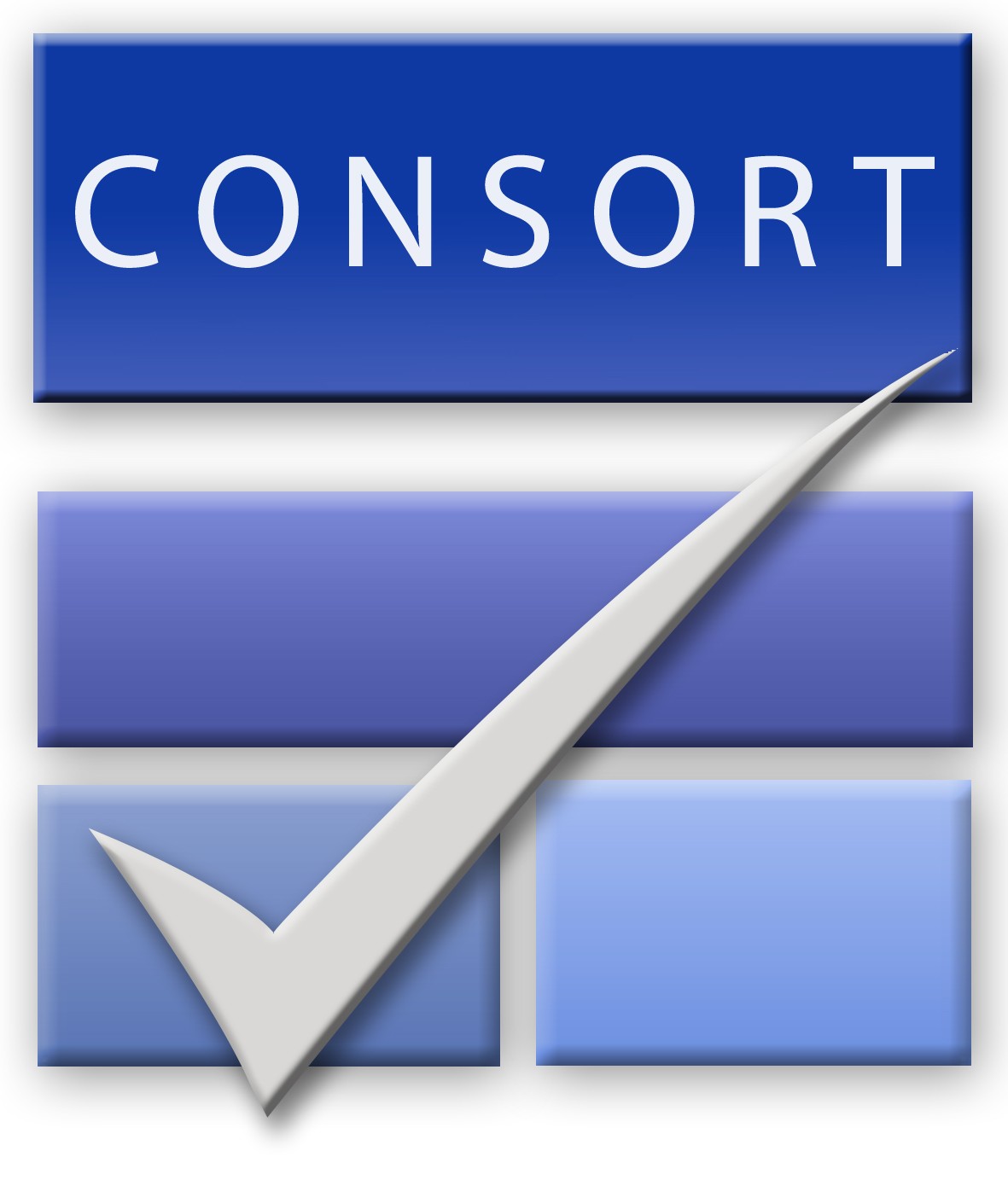
Supplementary Table 1 CONSORT 2010 checklist of information to include when reporting a randomised trial*

| Section/Topic | Item No | Checklist item | Reported on page No |
| --- | --- | --- | --- |
| Title and abstract | | | |
|  | 1a | Identification as a randomised trial in the title | 1 |
|  | 1b | Structured summary of trial design, methods, results, and conclusions (for specific guidance see CONSORT for abstracts) | 2 |
| Introduction | | | |
| Background and objectives | 2a | Scientific background and explanation of rationale | 2/3 |
|  | 2b | Specific objectives or hypotheses | 3 |
| Methods | | | |
| Trial design | 3a | Description of trial design (such as parallel, factorial) including allocation ratio | 3 |
|  | 3b | Important changes to methods after trial commencement (such as eligibility criteria), with reasons | N/A |
| Participants | 4a | Eligibility criteria for participants | Page 3 & Supp Table 2 |
|  | 4b | Settings and locations where the data were collected | 4 |
| Interventions | 5 | The interventions for each group with sufficient details to allow replication, including how and when they were actually administered | 4/5 |
| Outcomes | 6a | Completely defined pre-specified primary and secondary outcome measures, including how and when they were assessed | 6,7 |
|  | 6b | Any changes to trial outcomes after the trial commenced, with reasons | 6 |
| Sample size | 7a | How sample size was determined | 7/8 |
|  | 7b | When applicable, explanation of any interim analyses and stopping guidelines | N/A |
| Randomisation: |  |  |  |
| Sequence generation | 8a | Method used to generate the random allocation sequence | 4 |
|  | 8b | Type of randomisation; details of any restriction (such as blocking and block size) | 4 |
| Allocation concealment mechanism | 9 | Mechanism used to implement the random allocation sequence (such as sequentially numbered containers), describing any steps taken to conceal the sequence until interventions were assigned | 4 |
| Implementation | 10 | Who generated the random allocation sequence, who enrolled participants, and who assigned participants to interventions | 4 |
| Blinding | 11a | If done, who was blinded after assignment to interventions (for example, participants, care providers, those assessing outcomes) and how | 8 |
|  | 11b | If relevant, description of the similarity of interventions | 4/5 |
| Statistical methods | 12a | Statistical methods used to compare groups for primary and secondary outcomes | 7 |
|  | 12b | Methods for additional analyses, such as subgroup analyses and adjusted analyses | 7/8 |
| Results | | | |
| Participant flow (a diagram is strongly recommended) | 13a | For each group, the numbers of participants who were randomly assigned, received intended treatment, and were analysed for the primary outcome | 8 & figure 1 |
|  | 13b | For each group, losses and exclusions after randomisation, together with reasons | 8 & figure 1 |
| Recruitment | 14a | Dates defining the periods of recruitment and follow-up | 8 |
|  | 14b | Why the trial ended or was stopped | 8 |
| Baseline data | 15 | A table showing baseline demographic and clinical characteristics for each group | Table 1 |
| Numbers analysed | 16 | For each group, number of participants (denominator) included in each analysis and whether the analysis was by original assigned groups | All tables |
| Outcomes and estimation | 17a | For each primary and secondary outcome, results for each group, and the estimated effect size and its precision (such as 95% confidence interval) | All results |
|  | 17b | For binary outcomes, presentation of both absolute and relative effect sizes is recommended | N/A |
| Ancillary analyses | 18 | Results of any other analyses performed, including subgroup analyses and adjusted analyses, distinguishing pre-specified from exploratory | N/A |
| Harms | 19 | All important harms or unintended effects in each group (for specific guidance see CONSORT for harms) | 12 |
| Discussion | | | |
| Limitations | 20 | Trial limitations, addressing sources of potential bias, imprecision, and, if relevant, multiplicity of analyses | 15 |
| Generalisability | 21 | Generalisability (external validity, applicability) of the trial findings | 15 |
| Interpretation | 22 | Interpretation consistent with results, balancing benefits and harms, and considering other relevant evidence | 14/15/16 |
| Other information | | |  |
| Registration | 23 | Registration number and name of trial registry | 2 |
| Protocol | 24 | Where the full trial protocol can be accessed, if available | 17 |
| Funding | 25 | Sources of funding and other support (such as supply of drugs), role of funders | 17 |

*We strongly recommend reading this statement in conjunction with the CONSORT 2010 Explanation and Elaboration for important clarifications on all the items. If relevant, we also recommend reading CONSORT extensions for cluster randomised trials, non-inferiority and equivalence trials, non-pharmacological treatments, herbal interventions, and pragmatic trials. Additional extensions are forthcoming: for those and for up to date references relevant to this checklist, see www.consort-statement.org

**Supplementary Table 2**: Eligibility criteria

| **Inclusion** | **Exclusion** |
| --- | --- |
| 1.Women with histologically confirmed breast cancer 2. Patients with advanced breast cancer, i.e. locally advanced disease that is not amenable to curative surgical resection or with metastatic disease 3. HER2 positive or negative 4. ER and/or PR positive or negative 5. If ER positive there is no restriction on the number of lines of previous endocrine therapy for ABC 6. Performance status 0 or 1 7. Predicted life expectancy ≥ 3 months 8. BMI ≥24 kg/m2 9. Expressing a wish to lose weight 10. Not already entered or planned to enter a trial of an investigational medicinal product (IMP) for this line of therapy 11. Age >18 (can be pre or post-menopausal) 12. Measurable or non-measurable disease by RECIST v1.1 13. Patients with brain or leptomeningeal metastases are eligible as long as all sites have been treated with radiotherapy (+/- surgery) with evidence of disease control at least 8 weeks after the last dose 14. Women in whom further endocrine therapy is planned after chemotherapy are eligible. The treating clinician must state what endocrine therapy is planned before chemotherapy is initiated 15. Women with thyroid dysfunction are eligible provided they are euthyroid and on a stable dose of thyroxine for the last 6 months | 1. Physical or psychiatric conditions which may reduce compliance to and the safety of diet or resistance exercise, e.g. 1.1. Serious digestive and/or absorptive problems, including active inflammatory bowel disease. 1.2. Psychiatric disorders or conditions, e.g. history of eating disorders, untreated major depression, psychosis, substance abuse, severe personality disorder. 1.3. Bone metastases at risk of pathological fracture or that would limit resistance exercise through pain in all three areas of the body that are covered by the resistance exercises (upper limbs, trunk and lower limbs). Metastases may be ok if other areas of the body can safely be exercised. 2. Medications affecting adiposity or muscle mass and function and energy intake e.g. continuous daily steroids for longer than 4 weeks (short term steroids with chemotherapy are acceptable) 3. Diabetics on insulin or sulphonylureas (glibenclamide, gliclazide, glimepiride, glipizide, tolbutamide) as they could experience hypoglycaemia on restricted days of the intermittent diet (diabetics treated with diet alone or with any other medication including metformin are eligible) 4. Greater than Day 15 of this course of chemotherapy. 5. Visceral metastases that, in the opinion of the treating clinician, would result in death within 3 months if no response was achieved with this line of chemotherapy 6. Symptomatic or uncontrolled brain or leptomeningeal metastases |

**Supplementary Table 3: Recruiting sites for the B-AHEAD 3 study and numbers recruited from each site**

| **Hospital name** | **Location** | **Number recruited** |
| --- | --- | --- |
| The Christie NHS Foundation Trust | Greater Manchester | 44 |
| The Royal Albert Edward Infirmary | Greater Manchester | 10 |
| Macclesfield District General Hospital | Cheshire | 5 |
| Derriford Hospital  University Hospitals Plymouth NHS Trust | Devon | 4 |
| Leighton Hospital, Mid-Cheshire Hospitals NHS Foundation Trust | Cheshire | 2 |
| North Manchester General Hospital  Manchester University Hospitals Foundation Trust | Greater Manchester | 1 |
| Royal Stoke University Hospital  University Hospitals of North-Midlands | Cheshire | 1 |
| Royal Oldham Hospital  Northern Care Alliance | Greater Manchester | 1 |
| Salford Royal Hospital  Northern Care Alliance | Greater Manchester | 0 |
| Royal Devon and Exeter Hospital, Exeter  Royal Devon University Healthcare NHS Foundation Trust | Devon | 0 |
| Tameside & Glossop Integrated Care NHS Foundation Trust | Greater Manchester | 0 |
| Royal Liverpool Hospital, Liverpool Hospitals NHS foundation Trust | Merseyside | 0 |
| Royal Blackburn and Burnley  East Lancashire Hospitals NHS Trust | Lancashire | 0 |

**Supplementary Table 4: TIDieR checklist**


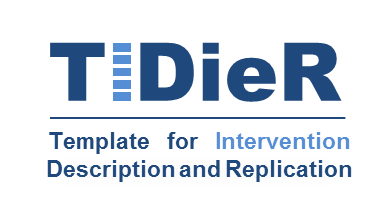
**The TIDieR (Template for Intervention Description and Replication) Checklist*:**

Information to include when describing an intervention and the location of the information

| **Item number** | **Item** | **Where located **** | |
| --- | --- | --- | --- |
|  |  | Primary paper  (page or appendix  number) | Other ^†^ (details) |
|  | **BRIEF NAME** |  |  |
| **1.** | Provide the name or a phrase that describes the intervention. | _________1___ | ______________ |
|  | **WHY** |  |  |
| **2.** | Describe any rationale, theory, or goal of the elements essential to the intervention. | _________1___ | _____________ |
|  | **WHAT** |  |  |
| **3.** | Materials: Describe any physical or informational materials used in the intervention, including those provided to participants or used in intervention delivery or in training of intervention providers. Provide information on where the materials can be accessed (e.g. online appendix, URL). | Supp table 5 / 7 | _____________ |
| **4.** | Procedures: Describe each of the procedures, activities, and/or processes used in the intervention, including any enabling or support activities. | __4/5__________ | _____________ |
|  | **WHO PROVIDED** |  |  |
| **5.** | For each category of intervention provider (e.g. psychologist, nursing assistant), describe their expertise, background and any specific training given. | _4, 5,6 _____ | _____________ |
|  | **HOW** |  |  |
| **6.** | Describe the modes of delivery (e.g. face-to-face or by some other mechanism, such as internet or telephone) of the intervention and whether it was provided individually or in a group. | _____4,5,6_____ | _____________ |
|  | **WHERE** |  |  |
| **7.** | Describe the type(s) of location(s) where the intervention occurred, including any necessary infrastructure or relevant features. | _______4,5,6______ | _____________ |
|  | **WHEN and HOW MUCH** |  |  |
| **8.** | Describe the number of times the intervention was delivered and over what period of time including the number of sessions, their schedule, and their duration, intensity or dose. | ________4,5,6__ | _____________ |
|  | **TAILORING** |  |  |
| **9.** | If the intervention was planned to be personalised, titrated or adapted, then describe what, why, when, and how. | ________5_____ | _____________ |
|  | **MODIFICATIONS** |  |  |
| **10.^ǂ^** | If the intervention was modified during the course of the study, describe the changes (what, why, when, and how). | _____N/A_____ | _____________ |
|  | **HOW WELL** |  |  |
| **11.** | Planned: If intervention adherence or fidelity was assessed, describe how and by whom, and if any strategies were used to maintain or improve fidelity, describe them. | _7___________ | _____________ |
| **12.^ǂ^** | Actual: If intervention adherence or fidelity was assessed, describe the extent to which the intervention was delivered as planned. | ___12__________ | _____________ |

** **Authors** - use N/A if an item is not applicable for the intervention being described. **Reviewers** – use ‘?’ if information about the element is not reported/not sufficiently reported.

† If the information is not provided in the primary paper, give details of where this information is available. This may include locations such as a published protocol or other published papers (provide citation details) or a website (provide the URL).

ǂ If completing the TIDieR checklist for a protocol, these items are not relevant to the protocol and cannot be described until the study is complete.

* We strongly recommend using this checklist in conjunction with the TIDieR guide (see *BMJ* 2014;348:g1687) which contains an explanation and elaboration for each item.

* The focus of TIDieR is on reporting details of the intervention elements (and where relevant, comparison elements) of a study. Other elements and methodological features of studies are covered by other reporting statements and checklists and have not been duplicated as part of the TIDieR checklist. When a **randomised trial** is being reported, the TIDieR checklist should be used in conjunction with the CONSORT statement (see [www.consort-statement.org](http://www.consort-statement.org/)) as an extension of **Item 5 of the CONSORT 2010 Statement.** When a **clinical trial** **protocol** is being reported, the TIDieR checklist should be used in conjunction with the SPIRIT statement as an extension of **Item 11 of the SPIRIT 2013 Statement** (see [www.spirit-statement.org](http://www.spirit-statement.org/)). For alternate study designs, TIDieR can be used in conjunction with the appropriate checklist for that study design (see [www.equator-network.org](http://www.equator-network.org/)).

**Supplementary Table 5**: Schedule of events for intervention delivery in the two study groups

| **Event** | **Timing** | **Location** | **Undertaken by** | **IER+RE** | **RE** |
| --- | --- | --- | --- | --- | --- |
| Dietary advice for the IER+RE group | Baseline | Prevent Breast Cancer Research Unit or called at home | B-AHEAD 3 dietitian | ✓ | 🗶 |
| Standardised guidance for managing side effects / food hygiene during chemotherapy | Baseline |  | Leaflet | ✓ | ✓ |
| Sent healthy tips sheets which covered key topics for adherence including, getting motivated, food & mood, shopping and eating out, protein, managing stress | 3 weekly during the IER+RE intervention | Received at home | Sent by B-AHEAD 3 dietitian |  |  |
| Resistance exercise advice | Baseline | Prevent Breast Cancer Research Unit or at local hospital | B-AHEAD 3 physiotherapist / cancer exercise specialist | ✓ | ✓ |
| Diet review calls | 3 weekly whilst in the trial | Patients called at home | B-AHEAD 3 dietitian | ✓ | 🗶 |
| Resistance exercise review calls | 3 weekly whilst in the trial | Patients called at home | B-AHEAD 3 physiotherapist / cancer exercise specialist | ✓ | ✓ |

**Supplementary Table 6**

Example of a low energy and low carbohydrate day and a Healthy Mediterranean diet day for the IER+RE group

| **Meal** | **Low energy and low carbohydrate day**  **2 consecutive days / week** | **Healthy Mediterranean diet**  **Other 5 days of the week for average participant in the trial height (1.6m) weight (76.5 Kg) age (58 years)** |
| --- | --- | --- |
| **Food portions**  Protein  Fat  Dairy  Vegetables  Fruit  Carbohydrate | Minimum 5 – maximum 9 (average 7)  Maximum 3  3  Minimum 5 – maximum 8  1  0 | 9 portions  Maximum 4  3  At least 5  2 fruit  Maximum 9 portions  Less than 10 units/ 80g of alcohol / week |
| **Breakfast** | Grilled mushroom and tomatoes with one scrambled egg | 2 slices wholemeal bread with 2 heaped tsp of peanut butter and 1 mashed banana |
| **Mid-Morning** | 1 piece fruit |  |
| **Lunch** | 1/2 tin of tuna and salad and oil dressing  Portion of walnuts | 120g / 4oz baked potato with ½ tub of cottage cheese  1 slice ham served with a mixed salad |
| **Mid-Afternoon** | Yoghurt, plain or diet | 10 cherry tomatoes/sliced pepper  2 tablespoon low fat hummus and 2 oatcakes |
| **Supper** | Celery sticks and low fat cream cheese | 2 tablespoons of unsalted nuts |
| **Other** | 1/3 pint milk in drinks throughout day | 1/3 pint milk in drinks throughout day |

**Supplementary Table 7: List of resistance exercises for the resistance exercise programme for both groups**

Examples of resisted exercises for the upper limbs

The number of repetitions or weight can be altered to suit the patient’s ability/performance status:

- Double arm press-ups against the wall in standing
- Single arms press-ups against the wall in standing
- Biceps arm curls with weight in standing or sitting
- Biceps arm curls followed by shoulder flexion with weight in standing or sitting
- Shoulder abduction to the ceiling with elbow in extension with weight
- Shoulder abduction with the elbow flexed with or without weight
- Shoulder circumduction backwards with or without weight
- Shoulder circumduction forwards
- Shoulder presses (i.e. using pectoralis major/minor) adduction and abduction with elbows and shoulders flexed to 90 degrees
- Shoulder extension against the wall

Examples of resisted exercises for the lower limbs

- Sitting to standing without use of hands
- Half squats holding onto the back of a chair
- Step up and down off a step
- Flex the knee to the chest in standing
- Abduct straight leg in standing

Examples of trunk exercises

- Side flexion to both sides with a weight in standing
- Trunk rotation with weight to both sides
- Stomach crunches in supine
- Stomach crunches diagonal in supine

**Supplementary Table 8: Schedule of assessments for both groups in the B-AHEAD 3** trial

| **Assessment** | **Timing** | **Location** | **Undertaken by** |
| --- | --- | --- | --- |
| **Primary objective** | | | |
| Progression free survival: CT scan read by local hospital | Routine scan, usually every 3-4 cycles or 9-12 weeks as per local guidelines | Local hospital | Organised by local oncology team |
| **Secondary objectives** | | | |
| Progression free survival: CT scan read centrally by trial radiologist | Routine scan, usually every 3-4 cycles or 9-12 weeks as per local guidelines | Local hospital | CT scan organised by local oncology team; images read centrally by trial radiologist |
| Time to Treatment Failure (TTF, i.e., time from randomisation to disease progression, death or discontinuation of therapy due to toxicity) | Recorded throughout study | Local hospital | Research nurse |
| Chemotherapy toxicity: self-reported CTCAE v4 (85) | Retrospective assessment of toxicity for previous cycle at the subsequent cycle of chemotherapy | Chemotherapy units | Research nurse records participant responses |
| Taxane neuropathy: assessed with automatic vibration assessment tool by identification of vibration/ no vibration on the index finger and big toe(VibraTip, McCallan Medical Ltd, Northamptonshire) | At each chemotherapy cycle (only participants on taxane chemotherapy) | Chemotherapy units | Research nurse |
| Body weight | At each chemotherapy cycle | Calibrated scales in chemotherapy units | Research nurse |
| Visceral and subcutaneous fat and lean body mass were assessed from the routine CT scan Visceral fat and muscle mass using commercial image analysis software (Analyze 12.0 - AnalyzeDirect Inc., Kansas, USA). | Routine scan, usually every 3-4 cycles or 9-12 weeks as per local guidelines | Local hospital | Organised by local oncology team |
| Lower and upper limb strength: five repetition sit to stand normalised by weight (79) and hand grip dynamometer tests | Baseline, then every 3 cycles | Prevent Breast Cancer Research Unit or chemotherapy units | Research nurse or B-AHEAD-3 researcher |
| Quality of life (FACT-B, FACT-ES FACT-F, FACT-BP FACT-Taxane for participants on taxane chemotherapy, hospital anxiety and depression score (HAD) | Baseline, start of cycle 3 then every 3 cycles after this | Chemotherapy units | Research nurse asks participant to complete questionnaires |
| Diet adherence for IER+RE group: self- reported record of low calorie days on a simple calendar | Recorded weekly whilst in the study in the IER | Home | Checked by B-AHEAD-3 researcher |
| Exercise adherence: self- reported record of resistance exercise sessions | Weekly for first 18 weeks of chemotherapy | Home | Checked by B-AHEAD-3 researcher |
| Physical activity assessment: seven day recall using the Scottish Physical Activity Questionnaire (Scot-PAQ) | Baseline, cycle 3 and cycle 6 | Home | Participant completes at home & e-mailed / posted back to the B-AHEAD-3 researchers |

**Supplementary Table 9: Change in leg & arm muscle strength from baseline (start of cycle 1) to cycle 3 (week 9)**

|  | **IER +RE (n=35)** | **RE(n=33)** | **All n=68** |
| --- | --- | --- | --- |
| Baseline leg strength | n=25 | n=23 | n=48 |
| Median (IQR) [range] | 10 (8.7-11.7) [6.7-21.9] | 11.4 (9.6-14.8) [5-25] | 10.6 (9-14) [5-25] |
| Cycle 3 Change in leg strength | n=12 | n=10 | n=22 |
|  | 0.4 (-0.9-1.5) [-7-35] | -0.3 (-1.3-0.5) [-3.2-12.8] | -0.2 (-1.3-1.3) [-7-35] |
| Baseline arm strength | n=33 | n=27 | n=60 |
| Median (IQR) [range] | 20.3 (15.3-23.2) [7.7-30.2] | 18 (12.7-23.5) [5.2-31.8] | 19.3 (13.5-23.4) [5.2-31.8] |
| Cycle 3 Change in arm strength | n=23 | n=19 | n=42 |
|  | 0.3 (-1.3-2.6) [-7.2-8.2] | 0.3 (-2.2-1.7) [-6.3-6.5] | 0.3 (-1.5, 1.8) [-7.2, 8.2] |
|  |  |  |  |

**Supplementary Table 10 Self-reported adherence to the resistance exercise programme and cardiovascular exercise in the two groups**

|  | **IER+ RE n=35** | **RE n=33** | **All n=68** |
| --- | --- | --- | --- |
| **Moderate/ vigorous cardiovascular activity (minutes/ week )*** |  |  |  |
| Baseline | n=31 | n=27 | n=58 |
| Median (IQR) [range] | 510 (202-788) [20-2235] | 510 (348-668) [10-1740] | 510 (270-718) [10-2235] |
| 9 weeks | n=25 | n=20 | n=45 |
| Median (IQR) [range] | 455 (270-780) [0-2430] | 338 (275-580) [70-2310] | 420 (270-735) [0-2430] |
| 18 weeks | n=16 | n=12 | n=28 |
| Median (IQR) [range] | 455 (168-791) [0-3240] | 675 (248-1162) [70-3000] | 585 (230-890) [0-3240] |
| 27 weeks | n=11 | n=6 | n=17 |
| Median (IQR) [range] | 540 (382-830) [280-3000] | 980 (532-1229) [200-2280] | 630 (390-1150) [200-3000] |
|  |  |  |  |
| **Resistance exercise (minutes / week)** |  |  |  |
| 9 weeks | n=24 | n=20 | n=44 |
| Median (IQR) [range] | 45 (0-90) [0-120] | 45 (23-95) [0-175] | 45 (0-90) [0-175] |
| 18 weeks | n=17 | n=11 | n=28 |
| Median (IQR) [range] | 60 (20-80) [0-120] | 30 (11-108) [0-280] | 53 (18-84) [0-280] |
| 27 weeks | n=11 | n=6 | n=17 |
| Median (IQR) [range] | 40 (15-81) [0-120] | 62 (47-106) [0-280] | 60 (30-100) [0-280] |

**Table 11. Time to treatment failure (progression or discontinuation of chemo)**

|  | **All n=68** | | **IER (n=35)** | | **Control (n=33)** | |
| --- | --- | --- | --- | --- | --- | --- |
|  | **n (%)** | **Follow-up (wks)** | **n (%)** | **Follow-up (wks)** | **n (%)** | **Follow-up (wks)** |
| **No progression** | 17 (25) | 8.7 (3.6, 23.9) | 9 (25.7) | 13.3 (3.6, 56.9) | 8 (24.2) | 8.4 (4.8, 17.2) |
| **Progression** | 46 (67.6) | 24.9 (10.6, 53.6) | 22 (62.9) | 25.1 (9.4, 52.4) | 24 (72.7) | 23.9 (11.9, 51) |
| **Discontinued Chemo** | 5 (7.4) | 20.6 (12, 21) | 4 (11.4) | 16.3 (11.3, 46) | 1 (3.1) | 21 |

| **HR (95%CI)** | **p-value (one-sided)** |
| --- | --- |
| **0.695 (0.389, 1.241)** | **0.11** |
| **0.75 (0.417, 1.348)** | **0.168** |
