# Supplementary material for: Randomised phase-2 screening trial of intermittent energy restriction plus resistance exercise versus resistance exercise alone during chemotherapy for advanced breast cancer
Source: Br J Cancer. 2025 Jul 31;133(7):1010–9. doi: 10.1038/s41416-025-03129-8 (PMC12479916; doi:10.1038/s41416-025-03129-8)
Supplement: Supplementary file 2 — supplemantary file [file 41416_2025_3129_MOESM2_ESM.pdf]

Supplementary Figure 1 Time to treatment failure for the IER+RE and RE groups

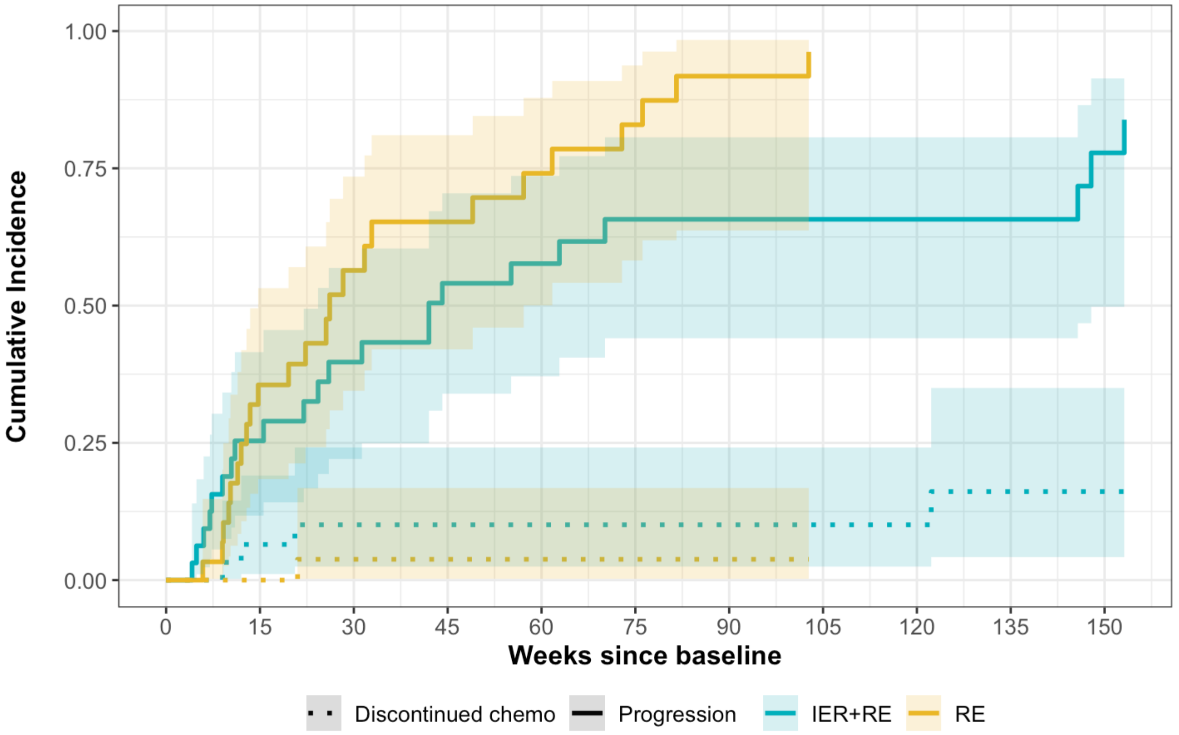

	0	15	30	45	60	75	90	105	120	135	150
**IER+RE**											
At Risk	35	19	14	10	8	6	6	4	4	3	1
Events	0	10	15	19	20	22	22	22	22	23	25
**RE**											
At Risk	33	18	9	7	5	3	1	0	0	0	0
Events	0	10	16	18	20	22	24	25	25	25	25

		HR (95%CI)	p-val (one-sided)
**Unadjusted**	**IER** (vs Control)	0.695 (0.389, 1.241)	0.11
**Adjusted**	**IER** (vs Control)	0.75 (0.417, 1.348)	0.168
